# Premenopausal Breast Cancer Risk Factors and Associations with Molecular Subtypes: A Case-Control Study

**DOI:** 10.1155/2021/5560559

**Published:** 2021-10-08

**Authors:** Faustin Ntirenganya, Jean Damascene Twagirumukiza, Georges Bucyibaruta, Belson Rugwizangoga, Stephen Rulisa

**Affiliations:** ^1^College of Medicine and Health Sciences, School of Medicine and Pharmacy, University of Rwanda, Rwanda; ^2^College of Medicine and Health Sciences, School of Public Health, University of Rwanda, Rwanda; ^3^Biostatistics, Spatio-Temporal Modeling of Infectious Diseases: Environment and Health Department, University of Waterloo, Canada

## Abstract

**Background:**

Breast cancer (BC) is the most prevalent cancer in women and the leading cause of women's cancer-related deaths and morbidity worldwide. In Rwanda, BC incidence is increasing with an unacceptably high mortality rate in premenopausal women.

**Objectives:**

The purpose was to identify modifiable BC risk factors and assess associations between common breast cancer risks factors and molecular subtypes in premenopausal women in Rwanda.

**Methods:**

This was a case-control study. Premenopausal women with histological confirmation of BC and frequency-matched for age controls were recruited. A preestablished questionnaire was administered to both cases and controls for sociodemographics, BC probable risk factors, and clinical and pathological characteristics. BC was classified into luminal A, luminal B, HER2-type, basal-like (triple negative), and unclassified molecular subtypes by immunohistochemistry (IHC). Odds ratio (OR) and 95% confidence interval (CI) were estimated using multivariate logistic regression analysis.

**Results:**

340 participants were recruited into the study (170 cases *vs.* 170 controls). The median age was 39 years. The majority of cases presented at advanced stages of the disease (51.2% in stages III and IV) and had invasive ductal carcinoma (98.2%). 60.6% had subtypes of poor prognosis (HER2 enriched 14.7%, triple negative 12.9%, and unclassified 32.9%). Alcohol intake (AOR = 3.73, 95%*CI* 2.19 − 6.32, *p* < 0.001), obesity/overweight in adolescence or early adulthood (AOR = 10.86, 95%*CI* 4.82 − 24.4, *p* < 0.001), history of primary infertility (AOR = 33.8, 95%*CI* 3.5 − 321.5, *p* = 0.002), nulliparity (AOR = 3.75, 95%*CI* 1.61 − 8.75, *p* = 0.002), and a history of benign breast disease (AOR = 6.06, 95%*CI* 1.19 − 30.73, *p* = 0.03) were associated with the occurrence of premenopausal breast cancer. There was no significant difference between risk factor stratification per molecular subtype.

**Conclusion:**

Several reproductive, environmental, and lifestyle risk factors have been identified to be associated with premenopausal BC. Among them, alcohol intake and obesity/overweight during adolescence/early adulthood can be modified. Interventions targeting alcohol consumption and obesity/overweight in adolescents and young adults may decrease the incidence of premenopausal breast cancer.

## 1. Introduction

Breast cancer is the most prevalent cancer and the leading cause of women's cancer-related deaths and morbidity worldwide. Although breast cancer is described as a disease of the elderly in developed countries, 50% of cases and 58% of deaths are occurring in developing countries within a relatively young population [1–4].

Indeed, breast cancer constitutes a major public health problem worldwide and remains a major scientific, clinical, and societal challenge generally in Africa and particularly in Rwanda. In Africa, publications on breast cancer describe a large number of patients presenting at a young age, with advanced disease and limited access to cancer education, screening, and care. We have learned from previous studies that registries are still scarce in Africa and few available data are generally from hospital-based logs. On the continent, accurate data are generally missing [4–7].

However, there are growing evidences suggesting that clinical presentation of breast cancer in African women may significantly be different compared to their counterparts in high-income countries. Breast cancer patients in Africa may be presenting at younger age and progress rapidly to advanced stages. In fact, the average age of breast cancer diagnosis seems to be 45 years or younger, a considerable difference compared to 64 and above of Caucasian populations [7–9].

Age structure difference alone may not entirely explain the younger age of breast cancer patients in Africa. There may be involved other additional factors to be determined. These may be genetic, molecular, hormonal, and environmental factors or interplay of them [10–12]. Unfortunately, it is not yet clear whether premenopausal breast cancers have different etiologic risk factors compared to postmenopausal BC. Indeed, the existing literature remains unclear or incomplete about the factors behind early-onset and rapid progression of breast cancer in African populations.

The purpose of this study was to identify modifiable risk factors and assess the associations between common risks factors and molecular subtypes in premenopausal breast cancer in Rwanda.

## 2. Methods

### 2.1. Study Design and Setting

This was a case-control study conducted at the University Teaching Hospital of Kigali (CHUK) and Butaro Cancer Center of Excellence (BCCOE) in Rwanda from September 2019 to September 2020.

#### 2.1.1. Population

Women attending breast clinics at CHUK and BCCOE have been recruited in the study. For the purpose of the study, the premenopausal period was defined based on self-rated menopausal status:

1. Any woman who reported having seen her menses within the last month was considered premenopausal as well as those who were pregnant or breastfeeding during the study period. In addition, women who reported not having seen menses in the previous 6 months for identifiable medical reasons (medications, diseases) were also considered premenopausal if aged ≤50 years
2. Any women who reported not having seen menses in the previous 6 months without any identifiable medical reasons (medications, diseases) were considered postmenopausal if aged ≥50 years. However, if aged ≤50 years, hormonal tests to determine menopausal status were conducted. High FSH levels (over 40 mIU/ml) and low estradiol levels (below 30 pg/ml) were diagnostic for premature menopause. Furthermore, regardless of their age, women who underwent bilateral surgical removal of ovaries were considered postmenopausal

#### 2.1.2. Study Participants' Selection

**Figure 1:**
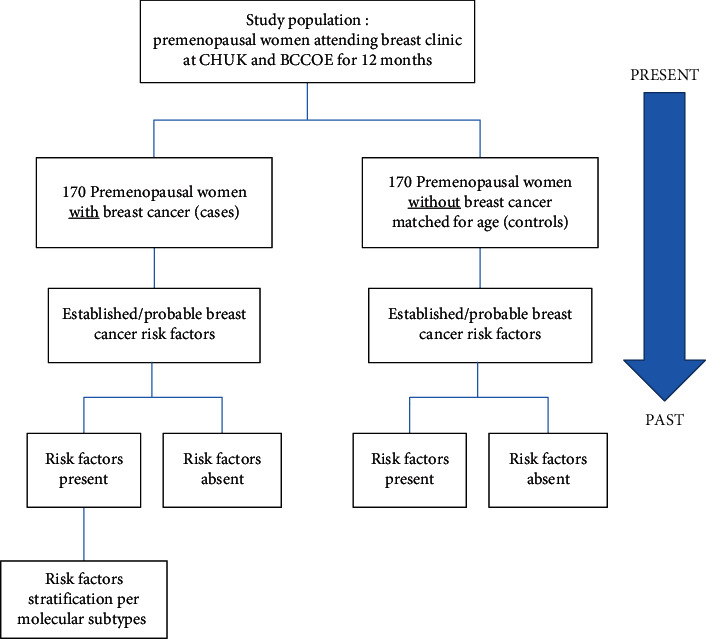
Methodology illustration.

Cases were women diagnosed with breast cancer in the premenopausal period. Potential cases were preidentified from outpatients' breast clinics and recruited in the study after histology confirmation of breast cancer and included in the study using enumerative sampling technique. For 12 months, consecutive cases of premenopausal breast cancers have been included in the study (Figure 1).

As far as controls are concerned, we used a 1 : 1 ratio. Women attending the breast clinic either for breast cancer screening or any other confirmed benign breast complaints were used as controls after clinical evaluation and imaging done to investigate their breast complaints was found to be normal or benign.

Controls were recruited on a weekly basis from the same breast clinic as cases, using stratified simple random sampling technique matching the age of cases recruited the previous week.

#### 2.1.3. Data Collection

A preestablished questionnaire was used to collect sociodemographic and potential risk factors for breast cancer. A data capture sheet was developed based on different questionnaires used in previous studies, and information on established or probable breast cancer risk factors (lifestyle, reproductive, hormonal, genetic, and medical history) was collected both for cases and controls. Clinical, histopathological, and immunohistochemistry findings were collected for cases using direct patients' interviews, pathology registries, reports, and patients' files.

#### 2.1.4. Sample Size Calculation

The sample size was calculated using G∗Power 3.1.9.7 for Windows, online software for sample size calculation, assuming 85% power, minimum odds ratio to detect of 2.0, percentage of exposed controls of 30%, alpha risk of 5%, 1 : 1 ratio. Therefore, the sample size was found to be 322 individuals with 161 cases and 161 controls.

### 2.2. Statistical Analysis

Data analysis was done using SPSS version 25.0 (IBM Corporation, New York 10504-1722, USA). Univariate analysis was conducted to compare sociodemographic characteristics of cases and controls. The Chi-squared test was used for categorical variables and nonparametric tests (Mann-Whitney *U* test) for continuous variables. Bivariate and multivariate logistic regression analysis was done for associations between risk factors and premenopausal breast cancer. Risk of premenopausal breast cancer was estimated by the odds ratio. For all odds ratios, 95% confidence interval was considered. A *p* value less than 0.05 was considered statistically significant.

### 2.3. Ethical Considerations

The study was approved by the IRB of the College of Medicine and Health Sciences, University of Rwanda, and by ethical committees of the CHUK and BCCOE. Written informed consent was obtained for both cases and controls prior to prospective data collection.

## 3. Results

348 participants were recruited in the study. 345 of them met inclusion criteria. Three cases and 2 controls had multiple missing data and were excluded. For that reason, 340 (170 cases and 170 controls) were retained for final analysis.

The median age of participants was 39 years with 45.9% of them aged below 40 years. Painless breast lump was the presenting sign in 45.6% of cases. 54.7% of patients reported intermediate to rapid progression of the disease (Table 1).

In bivariate logistic regression analysis, the crude odds ratio (COR) was calculated. Alcohol intake (COR = 4.49, 95% CI 2.82-7.16, *p* < 0.001), obesity/overweight in adolescence or early adulthood (COR = 12.8, 95% CI 6.13-26.8, *p* < 0.001), history of primary infertility (COR = 16.3, 95% CI 2.13-125.2, *p* = 0.007), history of benign breast disease (COR = 10.5, 95% CI 2.42-46.1, *p* = 0.003), nulliparity (COR = 2.18, 95% CI 1.18-4.04, *p* = 0.012), and contraceptive use (COR = 2.89, 95% CI 1.85-4.5, *p* < 0.001) were found to be associated with the occurrence of premenopausal breast cancer (Table 2).

Holding other relevant variables constant, the adjusted odds ratio was calculated in a multivariate regression analysis. Alcohol intake (*AOR* = 3.73, 95%*CI* 2.19 − 6.32, *p* < 0.001), obesity/overweight in adolescence or early adulthood (*AOR* = 10.86, 95%*CI* 4.82 − 24.4, *p* < 0.001), history of primary infertility (*AOR* = 33.8, 95%*CI* 3.5 − 321.5, *p* = 0.002), nulliparity (*AOR* = 3.75, 95%*CI* 1.61 − 8.75, *p* = 0.002), and a history of benign breast disease (*AOR* = 6.06, 95%*CI* 1.19 − 30.73, *p* = 0.03) were retained in the final fitting model as predictors of premenopausal breast cancer (Table 3).

By stratifying risk factors by molecular subtypes, there was no significant difference between risk factors stratified per molecular subtype (Table 4).

Invasive ductal carcinoma was the main histology type in 98.2% of patients while invasive lobular carcinoma and ductal carcinoma in situ represented 0.6% each (Table 5).

The most frequent molecular subtype was luminal A with 26.5% with subtypes of poor prognosis in 60.6% (HER2 enriched 14.7%, triple negative 12.9%, and unclassified 32.9%) (Table 6).

## 4. Discussion

This study identified risk factors for premenopausal breast cancer in Rwanda and stratified them per molecular subtypes.

Indeed, the majority of patients with breast cancer in Rwanda and in sub-Saharan Africa may be young premenopausal women, presenting with advanced stages of the disease and having poor outcomes [6, 8, 13–15]. The reasons why it happens like that are not yet fully understood. Marie Swanson et al. clearly demonstrate the existence of differences in age-specific incidence, risk factors, and outcomes, when comparing young African-Americans and White-Americans [10]. However, what they do not explain is why these epidemiological differences exist.

In our study, the median age of participants was 39 years with 45.9% of them aged below 40 years, aligning with previous studies [14, 16, 17]. On the one hand, population age structure and younger populations in Rwanda and African countries may explain partially the findings. However, age structure may give a false impression that breast cancer patients in Africa are predominantly young [18–21]. On the other hand, breast cancer heterogeneity may also support the hypothesis of BC younger age and aggressive presentation in premenopausal women as seen in this study. Indeed, breast cancer is not a single disease; it is rather a heterogeneous disease with different molecular subtypes behaving differently in clinical presentation, progression, and outcome [12, 22, 23]. Apparently, more aggressive breast cancer subtypes like triple negative or HER2/Neu-enriched tumors are mainly found in black populations and may present early [24–27].

Generally, premenopausal breast cancer is underdocumented. So far, the majority of available studies on breast cancer are conducted on postmenopausal breast cancers and suggest that premenopausal breast cancer may share the same risk factors with postmenopausal breast cancer [28]. However, there are growing evidences that premenopausal breast cancer may be having different risk factors. For example, extremely dense breasts and having a family history of breast cancer appear to be increasing specifically the risk of premenopausal breast cancers [28–30]. Furthermore, obesity and pauciparity seem to have no effect on premenopausal breast cancer while they are found to be increasing postmenopausal breast cancer [31, 32]. However, contrary to the latter findings, our study found an association between history of obesity/overweight in adolescence and early childhood and the developing breast cancer in the premenopausal period.

While our study found no differences in risk factor distribution among different molecular subtypes in premenopausal women with breast cancer, studies on etiologic heterogeneity of breast cancer linking environmental exposures to somatic mutations caused by smoking, exposures to infectious agents, or exposure to other known carcinogens supported the existence of distinct epigenetic profiles of breast cancer in general [22, 25, 33]. In addition, many studies have suggested that estrogen positive premenopausal breast cancer has distinct molecular characteristics compared to postmenopausal cancers and behaves differently with distinct integrin/laminin and EGFR signalling pathways [34–36]. Further studies on this subject are still needed.

Indeed, there are not yet enough evidences to conclude that premenopausal breast cancer is totally different to justify specific treatment guidelines, screening, and early detection strategies [37–39]. Breast cancer heterogeneity has been documented not only among different patients (intertumor heterogeneity) but also within each individual tumor (intratumor heterogeneity). The existence of different molecular subtypes of breast cancer indicating intratumor heterogeneity creates diagnostic and therapeutic challenges but has improved the classification of breast cancer patients into the low, intermediate, and high risk groups for personalized treatments [36, 40–43].

In our study, the majority of molecular subtypes identified in premenopausal women are estrogen negative molecular subtypes (basal-like and HER2/Neu-enriched). Even if young women with breast cancer are more likely to have genetic predisposition with BRCA1 and BRCA2 mutations, the expression of key biomarkers ER, PR, and HER2/Neu and proliferation markers like Ki67 appears to be different compared to postmenopausal cancers, confirming the above findings [11, 44, 45].

This study had some limitations. As in all case-control studies, we attempted to find correlations between past events and current status. Hence, due to its retrospective nature, there is room for potential recall bias, as there is an increased likelihood that those with outcomes will recall and report the exposure better compared to controls due to subject imperfect memories of past exposure. Furthermore, we may have failed to identify all confounding variables as there is no exhaustive list of probable risk factors for premenopausal breast cancer.

Lastly, we have not been able to conduct further analysis for equivocal HER2/Neu results. In fact, FISH technology is not available in the country. For the purpose of this study, tumors with equivocal HER2/Neu status were considered “unclassified”; this may have increased the number of unclassified tumors.

## 5. Conclusion

This study identified risk factors for premenopausal breast cancer in Rwanda and stratified them per molecular subtypes. Among the identified risk factors, alcohol intake and obesity/overweight during adolescence/early adulthood can be modified. Interventions targeting alcohol intake and obesity/overweight in young women may decrease the incidence of premenopausal breast cancer. Large-scale studies are still needed, to define whether premenopausal breast cancer is totally different from postmenopausal BC, to justify specific treatment guidelines, screening, and early detection strategies.

## Figures and Tables

**Table 1:** Sociodemographic and clinical characteristics of study participants (cases and controls, *N* = 340).

Variables	Cases (*N* = 170)		Controls (*N* = 170)	
	*n*	%	*n*	%
Age				
Median (Min–Max)	41 (18-50) years		35 (24-49) years	
Category				
<30 years	6	3.5	6	3.5
30-40 years	72	42.4	72	42.4
>40 years	92	54.1	92	54.1
Education				
No formal education	4	2.4	33	19.4
Primary	75	44.1	68	40
Secondary	77	45.3	60	35.3
University	14	8.2	9	5.3
Ubudehe (economic) category				
Ubudehe 1	9	5.3	7	4.1
Ubudehe 2	39	22.9	81	47.6
Ubudehe 3	119	70.0	81	47.6
Ubudehe 4	3	1.8	1	0.6
Profession				
Farmer	92	54.1	106	62.4
Business	47	27.6	38	22.4
Civil servant	16	9.4	11	6.5
Other	15	8.8	15	8.8
Family history of breast cancer				
Yes	33	19.4	23	13.5
No	137	80.6	147	86.5
Presenting symptoms				
Painless breast lump	155	45.6		
Breast pain	121	35.6		
Breast swelling	18	5.3		
Breast discharge	15	4.4		
Clinical stage				
Stage 1	5	2.9		
Stage 2	78	45.9		
Stage 4	63	37.1		
Stage 4	24	14.1		
Disease progression				
Slow progression	13	7.6		
Intermediate	93	54.7		
Rapid	64	37.6		

^∗^Ubudehe category version 2015: the category number increases with the higher socioeconomic status (government of Rwanda, community-led Ubudehe categorization) http://www.gov.rw/news_detail/?tx_ttnews[tt_news] =1054&cHash=a).

**Table 2:** Associations between exposure and premenopausal breast cancer.

Risk factor		Premenopausal breast cancer		COR	95% CI	*p*
		Cases (*n* = 170)	Controls (*n* = 170)			
Sports activity	Yes	157 (92.4%)	160 (94.1%)	0.75	0.32-1.77	0.518
	No	13 (7.6%)	10 (5.9%)			

Alcohol intake	Yes	100 (58.8%)	41 (24.1%)	4.49	2.82-7.16	<0.001
	No	70 (41.2%)	129 (75.9%)			

Obesity/overweight in adolescence/early adulthood	Yes	71 (41.8%)	9 (5.3%)	12.8	6.13-26.8	<0.001
	No	99 (58.2%)	161 (94.7%)			

Menstrual cycle regularity	Regular	77 (45.3%)	79 (46.5%)	0.95	0.62-1.46	0.828
	Irregular	93 (54.7%)	91 (53.5%)			

Menses quantity	Normal	129 (75.9%)	154 (90.6%)	0.32	0.17-0.60	<0.001
	Heavy	41 (24.1%)	16 (9.4%)			

History of primary infertility	Yes	15 (8.8%)	1 (0.6%)	16.3	2.13-125.2	0.007
	No	155 (91.2%)	169 (99.4%)			

Nulliparity	Yes	152 (89.4%)	135 (79.4%)	2.18	1.18-4.04	0.012
	No	18 (10.6%)	35 (20.6%)			

Contraception use	Yes	93 (54.7%)	50 (29.4%)	2.89	1.85-4.53	<0.001
	No	77 (45.3%)	120 (70.6%)			

History of benign breast disease	Yes	19 (11.2%)	2 (1.2%)	10.5	2.42-46.1	0.002
	No	151 (88.8%)	168 (98.8%)			

Family history of breast cancer	Yes	33 (19.4%)	23 (13.5%)	1.53	0.86-2.75	0.146
	No	137 (80.6%)	147 (86.5%)			

Radiation exposure	Yes	36 (21.2%)	42 (24.7%)	0.81	0.49-1.35	0.439
	No	134 (78.8%)	128 (75.3%)			

COR = crude odds ratio.

**Table 3:** Predictors of breast cancer in premenopausal women.

Predictor	Category	*z*	AOR	95% CI	*p*
Alcohol intake	Yes	4.88	3.73	2.19-6.32	<0.001
Obesity/overweight in past	Yes	5.76	10.86	4.82-24.4	<0.001
History of primary infertility	Yes	3.07	33.8	3.5-321.5	0.002
Nulliparity	Yes	3.07	3.75	1.61-8.75	0.002
History of benign breast disease	Yes	2.18	6.06	1.19-30.73	0.03

AOR: adjusted odds ratio.

**Table 4:** Associations between risk factors and molecular subtypes.

Risk factor	Luminal A (*n* = 45)		Luminal B (*n* = 22)		HER2 enriched (*n* = 25)		Triple negative (*n* = 22)	
	OR (95% CI)	*p*	OR (95% CI)	*p*	OR (95% CI)	*p*	OR (95% CI)	*p*
Sports activity								
Yes	2.07 (0.44-9.74)	0.355	1.24 (0.25-6.03)	0.785	2.16 (0.26-17.4)	0.468	1.74 (0.31-9.62)	0.526
No								
Alcohol intake								
Yes	1.79 (0.87-3.70)	0.112	1.26 (0.49-3.19)	0.624	1.05 (0.44-2.51)	0.897	1.29 (0.50-3.36)	0.591
No								
Obesity/overweight in past								
Yes	1.25 (0.62-2.52)	0.527	0.47 (0.17-1.29)	0.146	0.74 (0.31-1.74)	0.495	2.35 (0.91-6.07)	0.077
No								
Menstrual cycle regularity								
Regular								
Irregular	1.07 (0.54-2.13)	0.829	1.92 (0.74-4.99)	0.179	1.06 (0.45-2.49)	0.888	1.06 (0.41-2.73)	0.899
Menses quantity								
Normal								
Heavy	0.60 (0.25-1.42)	0.249	0.82 (0.30-2.27)	0.826	0.78 (0.30-2.04)	0.624	0.99 (0.32-3.01)	0.992
History of infertility								
Yes	1.01 (0.30-3.35)	0.986	0.55 (0.14-2.16)	0.399	0.39 (0.04-3.10)	0.374	—	
No								
Nulliparity								
Yes	1.2 (0.40-4.15)	0.666	2.12 (0.63-7.17)	0.224	1.78 (0.46-6.92)	0.399	1.08 (0.21-5.41)	0.921
No								
Contraception use								
Yes	1.98 (0.99-3.96)	0.052	1.01 (0.41-2.47)	0.987	240 (0.94-6.08)	0.066	1.05 (0.41-2.67)	0.914
No								
History of benign breast disease								
Yes	2.05 (0.56-7.41)	0.271	0.76 (0.20-2.88)	0.695	0.31 (0.10-0.91)	0.035^∗^	0.66 (0.13-3.22)	0.614
No								
Family history of breast cancer								
Yes	3.09 (1.02-9.37)	0.045^∗^	0.45 (0.16-1.23)	0.121	0.52 (0.14-1.86)	0.317	0.61 (0.19-1.19)	0.399
No								

**Table 5:** Histology type distribution (*n* = 170).

Histology type	*n*	%
Invasive ductal carcinoma	167	98.2
Invasive lobular carcinoma	1	0.6
Ductal carcinoma in situ	1	0.6
Other	1	0.6

**Table 6:** Prevalence of different breast cancer molecular subtypes (*n* = 170).

Molecular subtype classification	*n*	%
Luminal A	45	26.5
Luminal B	22	12.9
HER2 enriched	25	14.7
Triple negative	22	12.9
Unclassified^∗^	56	32.9

Luminal A: ER+/PR+, HER2/Neu negative; luminal B: ER+/PR+, HER/Neu positive; HER2 enriched: ER-/PR-, HER2/Neu positive; triple negative: ER-/PR-, HER2/Neu negative. ^∗^Unclassified: any other combination and equivocal HER2/Neu. It is important to note that for the purpose of this study, tumors with equivocal HER2/Neu status were considered “unclassified.” In fact, if the IHC result is 3+, the cancer is HER2/Neu positive. If the IHC result is 1+, the cancer is HER2/Neu negative. However, if the result is 2+, the HER2/Neu status is not clear (equivocal) and needs further testing by FISH to clarify the result. Unfortunately, FISH technology is not available in the country.

## Data Availability

The datasets used during the current study are available from the corresponding author on request.
